# Evolving Role of Risk Tailored Therapy in Early Stage HER2-Positive Breast Cancer: A Canadian Perspective

**DOI:** 10.3390/curroncol29060329

**Published:** 2022-06-06

**Authors:** Sharon F. McGee, Mark Clemons, Marie-France Savard

**Affiliations:** 1 Division of Medical Oncology, Department of Medicine, The Ottawa Hospital, The University of Ottawa, Ottawa, ON K1H 8L6, Canada; shmcgee@toh.ca (S.F.M.); mclemons@toh.ca (M.C.); 2 Cancer Therapeutics Program, Ottawa Hospital Research Institute, Ottawa, ON K1Y 4E9, Canada

**Keywords:** HER2-positive breast cancer, adjuvant, neoadjuvant, de-escalation

## Abstract

The advent of HER2-targeted therapies has led to an important shift in the management of HER2-positive early breast cancer. However, initial treatment approaches apply uniform treatment regimens to all patients, with significant treatment-related and financial toxicities for both the patient and the health care system. Recent data demonstrates that for many patients, the chemotherapy backbone, duration and nature (mono- versus dual-targeted therapy) of the HER2 blockade can be better targeted to an individual patient’s risk of recurrence. We will provide a review of current data supporting risk tailored therapy in early stage HER2-positive breast cancer along with key completed and ongoing Canadian and international risk tailored trials. Neoadjuvant systemic therapy should now be considered for patients with clinical stage 2 disease, with greater use of non-anthracycline based chemotherapy regimens. Patients with residual disease following neoadjuvant therapy should be considered for escalated treatment with adjuvant T-DM1. Patients with stage I disease can often be managed with upfront surgery and evidence-based de-escalated adjuvant chemotherapy regimens. The modest benefit of 12- versus 6 months of adjuvant HER2 therapy and/or dual adjuvant HER2 therapy should be carefully weighed against the toxicities. All patients with HER2-positive breast cancer should be enrolled in ongoing risk tailored treatment trials whenever possible. Increasing data supports risk tailored therapy in early stage HER2-positive breast cancer in place of the routine application of aggressive and toxic systemic therapy regimens to all patients. While much progress has been made towards treatment de-escalation in appropriate patients, more is needed, as we highlight in this review. Indeed, Canadian-led clinical trials are helping to lead these efforts.

## 1. Introduction

**Figure 1:**
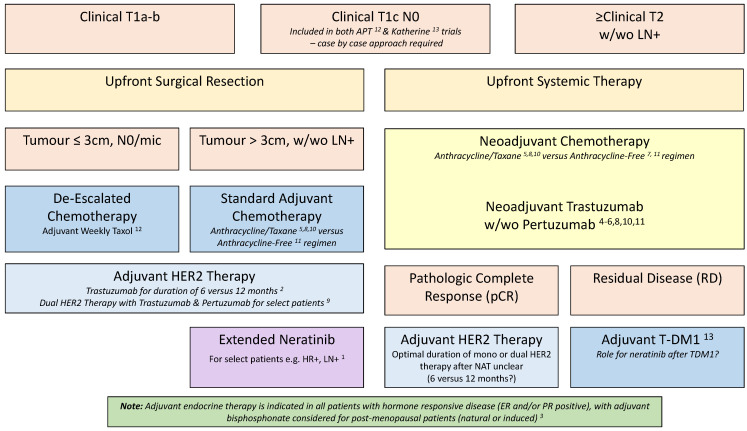
Current evidence-based risk tailored approach to HER2-positive EBC. EBC: early breast cancer; ER: estrogen receptor; HR: hormone receptor; LN: lymph node; NAT: neoadjuvant therapy; PR: progesterone receptor; w/wo: with/without. References: ^1^ [19]; ^2^ [19]; ^3^ [43]; ^4^ [32]; ^5^ [3]; ^6^ [5]; ^7^ [43]; ^8^ [1]; ^9^ [6]; ^10^ [2]; ^11^ [4]; ^12^ [21]; ^13^ [47].

Fifteen to twenty percent of patients with early breast cancer (EBC) will have over-expression of Human Epidermal Growth Factor Receptor-2 (HER2/ERBB2), an aggressive breast cancer subtype with a classically poorer prognosis. However, outcomes for these patients have improved significantly since the introduction of HER2 targeted therapy and have continued to improve as our knowledge of the biology of the disease increases. However, these benefits have come with considerable treatment-related and financial toxicities. In this review, we report advances in the treatment of HER2-positive EBC and the increasing move towards personalized, risk tailored strategies. We provide a practical guide for real-world clinical practice (Figure 1). We also provide updates on key ongoing clinical research in the field, with a focus on important Canadian studies.

## 2. Systemic Management of HER2-Positive EBC

### 2.1. Adjuvant HER2 Targeted Therapy

HER2 overexpression is predictive of response to HER2-targeted therapies, the prototype being the monoclonal antibody trastuzumab (Herceptin^®^ and multiple biosimilars). Adjuvant treatment with HER2-targeted therapy for a total of 12 months has been the standard of care for patients with HER2-positive EBC following data demonstrating that trastuzumab reduces the risk of recurrence by nearly a half and death by a third [1,2,3,4,5]. Higher risk patients (e.g., node-positive) may derive benefit from dual HER2 targeted therapy with trastuzumab and pertuzumab, with modest improvements (4.5%) seen in invasive disease-free survival (iDFS) in the APHINITY trial but no survival benefit to date [6]. Although approved by Heath Canada, adjuvant dual HER2 targeted therapy is not the standard of care in Canada, as the Canadian Agency for Drugs and Technologies in Health (CADTH) did not recommend its reimbursement.

The routine use of 12 months of HER2-targeted treatment has been questioned in a number of studies. Patients in the FinHer trial who received adjuvant trastuzumab for only 9 weeks and had similar outcomes to those seen in other adjuvant studies with longer durations of treatment [3,7,8]. To date, several trials have investigated shorter durations of HER2 targeted therapy, from 9 weeks to 6 months, with differing results [7,9,10,11,12]. Traditional meta-analyses have favored 12 months of adjuvant trastuzumab due to improved disease-free survival (DFS) and overall survival (OS) compared with shorter durations, despite the increased cardiac toxicity and costs [13,14,15,16]. However, a trend toward the decreased benefit of 12 months of therapy was noted for lower-risk patients [13]. This population may include patients with a lower disease burden, for example, tumor size < 2 cm and 0–3 lymph nodes involved, as suggested by subgroup analyses of the ShortHER study [17].

More recently, an individual patient data meta-analysis of five non-inferiority randomized controlled trials of reduced duration single agent adjuvant trastuzumab in HER2-positive EBC was presented [18]. This found that while iDFS with 9 weeks of trastuzumab was not non-inferior to 12 months, 6 months of trastuzumab was non-inferior to 12 months. Indeed, continuing to 12 months following the completion of 6 months of HER2 therapy provided a marginal 0.7% additional benefit in iDFS. Despite this, commonly used clinical practice guidelines have yet to endorse 6 months as a standard of care, highlighting the need for further research on the application of these data.

Finally, although the HERA trial showed no difference in disease outcomes with 1 versus 2 years of adjuvant trastuzumab [8], the EXTENET trial suggested a benefit for extended HER2 therapy in select patients [19]. In this study, patients were randomized to oral placebo or neratinib, a pan-HER tyrosine kinase inhibitor (TKI), for 1 year, within 2 years of completion of standard treatment with systemic chemotherapy and trastuzumab for 1 year [19]. Results showed improved iDFS in patients with HER2 and hormone receptor (HR) positive disease. In subgroups analysis, the benefit appeared more significant for patients with node-positive disease or those who started neratinib within 1 year of trastuzumab completion. Neratinib also reduced the incidence of central nervous system (CNS) recurrence. However, these benefits were limited by the 39% incidence of grade 3 diarrhea. It is important to note that diarrhea prophylaxis was not mandatory in this study. The subsequent CONTROL study showed that a dose-escalation strategy, starting at half-dose for 1 week, then two-thirds dose for 1 week and then the full dose, with loperamide as needed, significantly minimized the risk of diarrhea associated with neratinib [20]. However, the EXTENET study pre-dates the increased use of neoadjuvant pertuzumab and post-neoadjuvant antibody-drug conjugate T-DM1 (trastuzumab-emtansine). Thus, there is no data on the added benefit of neratinib in these specific populations. The CADTH review did not support neratinib reimbursement based on EXTENET data.

### 2.2. Adjuvant Chemotherapy in HER2-Positive Disease

The benefits of adjuvant HER2 therapy have been established in combination with a variety of chemotherapy backbones, with original trials favoring combination taxane and anthracycline chemotherapy [1,2,3,4]. The requirement for multiagent chemotherapy for all HER2-positive EBC patients was questioned in the seminal single-arm APT trial, which established weekly paclitaxel for 12 weeks with 1 year of trastuzumab, as the standard of care for lower risk HER2-positive disease, with tumors ≤ 3 cm and negative or microscopic nodal involvement [21].

The phase 2 ATEMPT trial compared adjuvant weekly paclitaxel for 12 weeks with 1 year of trastuzumab to 1 year of T-DM1 in patients with stage I HER2-positive EBC. Three-year invasive disease-free survival was similar between the groups (93.4% for paclitaxel-trastuzumab vs. 97.8% for T-DM1) [22]. The toxicity profiles were different, with alopecia, neurotoxicity, leukopenia, diarrhea and infusion reactions with paclitaxel, and more hyperbilirubinemia, thrombocytopenia and dose reductions in the T-DM1 arm [22]. Thus, adjuvant T-DM1 may be an option for patients wishing to avoid specific toxicities of paclitaxel chemotherapy. However, more evidence-based chemotherapy options for low risk HER2-positive EBC patients are needed. As far as we know, adjuvant T-DM1 in this setting is not approved in Canada.

The original adjuvant HER2 trials established anthracycline-based chemotherapy as the standard for patients with high-risk HER2-positive EBC [1,2,3]. This was challenged by Slamon and colleagues in the BCIRG-006 trial, which demonstrated similar disease outcomes with adjuvant trastuzumab, docetaxel and carboplatin, compared with trastuzumab and anthracycline-based chemotherapy (DFS 74.6% vs. 73%, OS 85.9% vs. 83.3%) [4,23]. There was, however, a significantly higher rate of grade 3/4 congestive heart failure with anthracycline-based chemotherapy (0.4% vs. 2%) and seven cases of leukemia, compared with none in the carboplatin arm [23]. While this trial was not designed or powered to compare the two trastuzumab containing regimens, it was the first important move toward anthracycline-free chemotherapy in HER2-positive EBC patients. Subsequently, in the APHINITY trial [6], this non-anthracycline containing regimen was allowed and used for 22% of the enrolled patients.

### 2.3. Neoadjuvant Therapy in HER2-Positive Disease

From the first preoperative chemotherapy studies, the National Surgical Adjuvant Breast and Bowel Project (NSABP) B-18 and B-27 trials, preoperative chemotherapy was shown to be equivalent to adjuvant chemotherapy [24,25]. Although initially reserved for patients with locally advanced or inflammatory breast cancer, neoadjuvant therapy (NAT) has become the standard for patients with clinical stage 2 disease, particularly those with HER2-positive and triple-negative breast cancer (TNBC) [26,27]. Here, NAT allows patients to be risk-stratified based on their response to upfront systemic therapy, where patients with residual disease at the time of surgery can benefit from additional systemic therapy due to their higher risk of recurrence and death. Patients who achieve a pathologic complete response (pCR) with no evidence of invasive disease in the breast and axillary surgical specimen have an excellent prognosis and may be considered for de-escalated treatment. Indeed, pooled analyses of patients treated with NAT have shown improved long-term outcomes for those who achieve a pCR [28,29], with recent data from the I-SPY Clinical Trials Consortium showing 5 and 10-year Event Free Survival (EFS) for HER2-positive EBC patients who achieve a pCR with NAT, at 93–94% and 90–91%, respectively [30]. Pathologic complete response has thus become an important NAT trial endpoint in HER2-positive EBC to support the accelerated approval of drugs. As the CTNeoBC study did not establish pCR as a trial-level surrogate marker but only as an individual-level surrogate marker, the approval of drugs by international regulatory agencies remains conditional to meaningful and statistically significant DFS, EFS or OS improvement in a confirmatory trial [31].

### 2.4. Neoadjuvant HER2 Targeted Therapy

Support for the use of dual HER2 targeted therapy in the neoadjuvant setting was primarily based on the results of the phase 2 NEOSPHERE trial, where neoadjuvant pertuzumab, trastuzumab and docetaxel improved pCR rates (45.8%, 49 of 107 patients) compared to trastuzumab plus docetaxel (29%, 31 of 107 patients), pertuzumab plus docetaxel (24%, 23 of 96 patients) and pertuzumab plus trastuzumab (16.8%, 18 of 107 patients) [32]. An important caveat that complicates the interpretation of this study is that anthracycline-containing chemotherapy was administered after surgery, which goes against the FDA guidance on using pCR as an endpoint to support accelerated approval [31]. There is no guarantee that this pCR difference would hold true if anthracycline was administered before the surgery. Although this study was not powered for long-term outcomes, the authors later published 5-year progression-free survival (PFS) rates, with an exploratory analysis showing improved PFS for patients who achieved pCR, irrespective of treatment assignment, compared to those with residual disease (85% vs. 76%, HR 0.54) [33]. Since the four treatment groups were pooled in this analysis, it does not definitely confirm that the pCR rate differences between treatment groups predict long term benefits. Based on this and other neoadjuvant phase II studies [34,35], Health Canada has approved the addition of pertuzumab to neoadjuvant trastuzumab and chemotherapy for patients with HER2-positive, locally advanced, inflammatory or EBC, either >2 cm or node-positive. However, the CADTH did not recommend pertuzumab for reimbursement, and, in Canada, its access is neither uniform nor standard.

Neoadjuvant dual HER2 targeted therapy with trastuzumab and short course lapatinib (12–16 weeks) has also been studied, with a recent meta-analysis of four trials [36,37,38,39] showing a relative decrease in the risk of relapse and death, of 38% and 35%, respectively, compared with trastuzumab alone [40]. This study is also the first to correlate an increase in pCR rates with improved outcomes, with patients achieving a pCR experiencing a relative reduction in the risk of relapse and death, of 55% and 45%, respectively [40]. However, lapatinib is not approved in the neoadjuvant setting in Canada.

### 2.5. Neoadjuvant Chemotherapy for HER2-Positive Disease

Neoadjuvant trials have provided further support for anthracycline-free chemotherapy in HER2-positive EBC. The largest of these studies was TRAIN2, which demonstrated equivalent pCR rates (67% vs. 68%), and 3-year EFS (92.7% vs. 93.6%) and OS (97.7% vs. 98.2%), with anthracycline-based (consisting of 5-fluorouracil, epirubicin, and cyclophosphamide every 3 weeks for three cycles followed by paclitaxel and carboplatin on day 1 and 8 every 3 weeks for six cycles) and non-anthracycline-based (nine cycles of paclitaxel and carboplatin on day 1 and 8 every 3 weeks) regimens, respectively [41,42]. As with BCIRG-006, subgroup analysis showed no evidence that patients with higher risk disease (large tumors, high grade, multiple nodes) derived more benefit from anthracyclines [4,42]. From a Canadian perspective, the two TRAIN2 regimens with their nine cycles of chemotherapy are not used in the standard practice, but the ones studied in BCIRG-006 are commonly administered. Overall, guidelines now support a move away from the routine use of anthracyclines in HER2-positive EBC, which has also been commonly incorporated into the Canadian practice [27,43].

However, studies are increasingly using de-escalated neoadjuvant chemotherapy for all patients with HER2-positive EBC, regardless of disease burden or clinical risk. For example, the Adjuvant Dynamic Marker-Adjusted Personalized Therapy (ADAPT) umbrella trial is investigating de-escalated systemic therapy across the four breast cancer subtypes [44]. In the HER2-positive/HR negative trial, patients with clinical T1-4 and N0-3 disease were randomized 5:2 to neoadjuvant trastuzumab and pertuzumab, with or without paclitaxel for 12 weeks, followed by surgery [45]. After surgery, treatment according to national standards was recommended (e.g., anthracycline/taxane-based chemotherapy and 40 weeks of trastuzumab). However, further chemotherapy could be omitted at the investigator’s discretion, which occurred for 79% of patients in the paclitaxel arm and 29% in the HER2 therapy alone arm [46]. Ninety percent of patients who received neoadjuvant paclitaxel and dual HER2 therapy achieved a pCR, with a 5-year iDFS and OS of 98% [45,46]. For patients treated with neoadjuvant dual HER2 therapy alone, 34.4% achieved a pCR, with iDFS and OS of 87% and 94% [45,46]. Approximately 70% of patients who achieved a pCR in the arm without neoadjuvant chemotherapy received further adjuvant chemotherapy making it challenging to know whether or not it was the pCR with HER2 therapy alone that led to the excellent prognosis in this subgroup of patients. Additional research is needed in this area, and several exciting de-escalation trials are ongoing.

### 2.6. Post-Neoadjuvant HER2 Targeted Therapy

Patients with residual HER2-positive disease following NAT have a poor prognosis due to a higher risk of future disease recurrence [28,30]. The seminal Katherine study established NAT as the standard of care for patients with HER2-positive EBC and ≥T1c, N0 clinical disease by demonstrating a significant improvement in iDFS for patients with residual disease who completed treatment with 14 cycles of T-DM1 following surgery [47]. The trial results showed a 50% reduction (corresponding to an 11% absolute reduction) in the risk of recurrent invasive breast cancer or death for patients who switched to T-DM1 compared with those who continued with standard treatment with trastuzumab for a total of a year [47]. In the interim, new HER2 antibody-drug conjugates have entered clinical use in the metastatic setting, with excellent results. As such, the results of the ongoing DESTINY-Breast 05 trial (NCT04622319) comparing TDM1 and TDXd (trastusumab-deruxtecan) in HER2-positive EBC patients with residual disease following NAT are eagerly awaited.

## 3. Ongoing Trials of Risk Tailored Therapy in HER2-Positive EBC

Given that pCR after NAT offers an in vivo assessment of the tumor biology and is an individual surrogate marker for long-term DFS, pCR could be used in identifying patients at lower or higher risk of relapse post-NAT to guide subsequent treatment [29,47]. This selection approach is being integrated into the design of new trials where a reduced NAT regimen is used to start, and the choice of additional therapies is made based on the response measured on the surgical specimen to spare some patients from unnecessary treatment toxicity.

### 3.1. Ongoing Trials Leveraging pCR as a Selection Approach for Adjuvant Therapy

The ongoing Decrescendo (NCT04675827) and CompassHER2-pCR (NCT04266249) trials [48] are multicenter, open-label, single-arm phase II de-escalation trials where all subjects will receive four cycles of NAT with a combination of taxane and dual HER2 blockade with trastuzumab and pertuzumab. If pCR is confirmed, 14 additional cycles of trastuzumab and pertuzumab will be administered. If pCR is not achieved, T-DM1 alone or after additional standard of care chemotherapy will be received. The primary endpoint of both trials is recurrence-free survival. The main difference between these two trials is their eligibility criteria. In the Decrescendo trial, only patients with clinically node-negative HER2-positive hormone receptor negative breast cancer with a tumor size between 15 and 50 mm will be enrolled. In the CompassHER2-pCR trial, clinically node-positive breast cancer patients can be included if T1–T3. Patients with T4 and N3 disease are excluded. Additionally, in the Decrescendo trial, a fixed dose of subcutaneous trastuzumab and pertuzumab are used instead of the standard intravenous formulation. Although both Decrescendo and CompassHER2-PCR attempt to de-escalate the chemotherapy backbone based on pCR, if we fully embrace this concept, one can question the benefit of completing a full year of anti-HER2 therapy when pCR is achieved. This critical question will be further explored in the important Canadian REaCT-HER TIME trial (details below).

The CompassHER2-pCR trial has an escalation study counterpart, CompassHER2-RD (NCT04457596). The latter is a multicenter phase III study where HER2-positive breast cancer patients with the residual disease are randomized to receive 14 cycles of T-DM1 plus tucatinib or a placebo. The inclusion criteria are broader than the CompassHER2-pCR ones and include patients with clinical stage T1–T3 and N0–N3 at presentation. The primary endpoint is modified iDFS. Among the secondary endpoints are brain metastasis DFS and incidence of brain metastasis. As tucatinib is known to penetrate the blood–brain barrier and has yielded impressive results in the treatment of brain metastasis with prolonged survival in the metastatic setting [49], it will be interesting to see if these benefits hold true in a non-metastatic setting.

In an effort to completely avoid chemotherapy side effects, a chemotherapy-free therapeutic approach is being studied in the PHERGAIN-2 trial (NCT 04733118) [48], where patients with clinical T > 5 to 25 mm and clinical N0 disease will receive eight, 3-week cycles of subcutaneous trastuzumab and pertuzumab. Depending on the pathological response, treatment with trastuzumab and pertuzumab will be continued for those with a pCR, while those with the residual disease will switch to T-DM1. Chemotherapy can be administered before adjuvant T-DM1 at the discretion of the treating physician. Endocrine therapy with letrozole for postmenopausal women or Tamoxifen with/without ovarian suppression for pre-menopausal women will be added in the neoadjuvant and adjuvant setting for HR-positive breast cancer. The primary outcomes are a 3-year recurrence-free interval and global health status decline.

### 3.2. Ongoing De-Escalation Studies

It is refreshing to see the beginning of global efforts to reduce the treatment burden by optimizing the chemotherapy backbone, shortening treatment duration and decreasing the number of visits by developing and using subcutaneous or oral formulations. Ongoing and active de-escalation studies for early-stage HER2-positive breast cancer were identified by searching ClinicalTrials.gov on 11 March 2022 and using the terms “HER2-positive breast cancer” and “de-escalation” (Table 1) [48]. Similar to DECRESCENDO and CompassHER2-pCR trials, the DAPHNe trial (NCT03716180) uses a reduced upfront NAT for stage 2 and 3 breast cancer. The trial aim is to evaluate the acceptability of a post-neoadjuvant treatment recommendation based on pCR, i.e., if pCR is achieved, no additional chemotherapy is recommended, but with continuation with trastuzumab and pertuzumab. Some studies showed that imaging such as PET scans and MRI or serial breast core needle biopsies during NAT could not predict pCR with enough accuracy to omit surgery [50,51]. As the MRI accuracy in predicting residual disease is greater for higher-grade tumors, in TRAIN-3 (NCT03820063), the benefit of a shorter duration of chemotherapy followed by an earlier surgery is being evaluated for breast cancer patients with an early complete radiologic response. The chair time and the increased number of visits with intravenous therapy are associated with a clinical and economic burden. In the DECRESCENDO trial, subcutaneous trastuzumab and pertuzumab are used instead of the intravenous form. Additionally, two phase 2 studies, IRIS (NCT04383275) and IRIS-C/D (NCT04383275), evaluate an oral chemotherapy regimen with either capecitabine or vinorelbine or endocrine therapy alone in combination with trastuzumab for lower-risk HER2-positive breast cancer.

## 4. Canadian-Led Pragmatic Trials to Optimize Standard of Care

Important Canadian work has been conducted to optimize practices by evaluating the standard of care interventions in both the supportive care and treatment setting. The ultimate goal is to reduce unnecessary treatment-related and financial toxicity and foster an evidence-based approach to treatment.

Considering the prolonged duration of intravenous treatment for HER2-positive breast cancer and the use of vesicant chemotherapy agents, different central venous access devices are often inserted for patients that may have so-called “difficult” veins or to prevent complications associated with peripheral vein access, such as extravasation, infiltration or phlebitis [52]. In REaCT (REthinking Clinical Trials)-Vascular Access (VA), patients with HER2-positive disease receiving trastuzumab-based neo/adjuvant chemotherapy had a similar rate of line access complications (e.g., infections, thromboembolism, extravasation, treatment delays due to vascular access, thrombolytic usage) whether they were randomized to a PICC or a PORT (17.2% vs. 14.8%) [53]. Interestingly, in the REaCT-VA HER2 negative study, patients were randomized to peripheral or central access, with no difference in the rate of complications (4.67% vs. 4.67%). However, almost 12% of patients randomized to peripheral access eventually required a central line [54].

Treatment-related cardiac toxicity has been an important concern in the HER2-positive breast cancer population because it might prevent patients from receiving further HER2-targeted therapy and ultimately affect outcomes as well as survivorship. As the optimal frequency of standard cardiac monitoring during trastuzumab-based therapy is based on little evidence [55], the REaCT-Ejection Fracture (EF) study compared 3 vs. 4-monthly cardiac imaging [56]. It confirmed that the 4-monthly left ventricular ejection fraction (LVEF) evaluation is non-inferior to the 3-monthly in terms of LVEF change throughout the course of trastuzumab-based therapy, rates of cardiac dysfunction, rates of delay and discontinuation of trastuzumab therapy, referrals to cardiology, the incidence of heart failure and emergency department visits [56].

LVEF dysfunction due to anti-HER2 targeted therapies such as trastuzumab and pertuzumab is mostly reversible as opposed to anthracycline-induced cardiotoxicity [57]. Therefore, even after a significant LVEF drop (i.e., >10% from baseline and below the upper normal limit), it might be appropriate to adopt a permissive approach to avoid holding anti-HER2 targeted therapy in a population subset that is minimally symptomatic and receiving appropriate cardioprotective medication. The SCHOLAR-2 trial will help answer this important question. SCHOLAR-2 is a multicenter, randomized trial that evaluates the safety and efficacy of continuing trastuzumab/pertuzumab/T-DM1 in patients with stage I-III HER2-positive breast cancer who develop systolic LV dysfunction defined as an LVEF < 54% or LVEF ≥ 54% and either a fall in LVEF of ≥15% from prior baseline or New York Heart Association class II heart failure symptoms within the past 6 months (NCT04680442) [48]. Patients with an LVEF of <40% and a New York Heart Association (NYHA) class III and IV heart failure are excluded. This trial is currently recruiting.

Two multicenter, open-label, de-escalation studies are currently being conducted by the REaCT program. The REaCT low-risk HER2 study (NCT03705429) compared two commonly used systemic therapy regimens for early-stage HER2-positive breast cancer to establish the optimal option in terms of toxicity profile, cost-effectiveness and quality of life measured with FACT-Taxane and -Fatigue scores [48]. In this trial, patients were randomized to four cycles of either docetaxel, cyclophosphamide and trastuzumab or weekly paclitaxel and trastuzumab, followed by trastuzumab maintenance. It is now closed to accrual, and results should be available soon.

Despite recent data supporting a shorter duration of adjuvant HER2 therapy as outlined above, there is persistent resistance to considering the 6-month duration of trastuzumab as a standard of care. Key barriers to its uptake include the fact that the trials conducted treated patients with an anthracycline regimen, which is no longer the standard of care regimen. Furthermore, the patient population of the trials driving the non-inferiority of 6 versus 12 months of treatment were largely lower risk, with approximately half of the patients being node-negative with tumors less than 2 cm [58,59]. Therefore, the question remains: will the non-inferiority of 6 months of trastuzumab hold true for higher clinical stage breast cancers and in patients not treated with an anthracycline? In the Canadian REaCT-HER TIME study, patients with HER2-positive breast cancer who achieved a pCR will complete a total of 6 months of adjuvant trastuzumab, regardless of their initial clinical stage or the chemotherapy backbone received (NCT04928261) [48]. This single-arm trial design is based on the evidence that pCR-selected patients have a better prognosis with a 5 and 10 yrs EFS above 90%. This trial design is similar to the single-arm APT trial that was deemed sufficient to change clinical practice for lower-risk breast cancer.

## 5. Discussion

The treatment of HER2-positive EBC has changed significantly since trastuzumab was first approved for use alongside systemic chemotherapy in 2006. Given the aggressive biology of the disease and poor patient prognosis, original treatment strategies followed a similarly aggressive approach. However, we increasingly see that for select patients, “less is often more”, with de-escalated treatment yielding excellent disease outcomes with fewer toxicities. More work is needed, however, to develop risk tailored management strategies for individual patients with HER2-positive EBC.

However, psychosocial factors represent an important barrier to the uptake of de-escalated treatment in clinical practice. These include the tendency for physicians to favor the status quo (entrenched behaviour), with our decisions often more influenced by potential losses than similarly sized gains (loss aversion) [18]. For this reason, it is increasingly important to involve patients in the decision-making process to determine their individual risk–benefit thresholds.

Innovative clinical trial platforms such as the Ottawa Hospitals REaCT (Rethinking Clinical Trials) program are increasingly important in this regard, as they provide patients with access to patient-centered, pragmatic clinical trials. In these studies, patients are involved in every stage of the process, from research and design, to conduct and dissemination. With several important de-escalation trials for HER2-positive EBC, the REaCT program will play a key role in advancing risk tailored therapy for HER2-positive EBC patients.

Alternative approaches to trial design are also necessary to expedite research findings. Traditional randomized controlled trials in smaller patient populations such as HER2-positive disease require larger sample sizes with prolonged recruitment and follow-up. In response, Tolaney and colleagues developed the single-arm APT trial, which recruited 400 patients with low-risk HER2-positive EBC and established de-escalated treatment with single-agent paclitaxel and trastuzumab as the standard of care for this population [21]. Therefore, a single-arm trial in HER2-positive EBC can quickly and effectively change clinical practice and is currently being pursued in several important ongoing clinical trials discussed here (DECRESENDO, CompassHER2-pCR, PHERGAIN-2, REaCT-HER TIME). The use of pCR as a surrogate endpoint is another important strategy to support accelerated drug approval and has led to the approval of pertuzumab by the FDA.

## 6. Conclusions

In this review, we provide a practical overview of current data supporting risk tailored treatment in HER2-positive EBC and ongoing studies in Canada and abroad. However, Canada can do more by conducting more risk-adapted trials for HER2-positive EBC, embracing evidence-based treatment de-escalation in clinical practice, and advocating for improved access to novel HER2 treatment strategies for Canadian patients.

## Figures and Tables

**Table 1:** Ongoing active de-escalation studies for curative treatment of HER2-positive breast cancer.

Trial Name, NCT Number and Sponsor	Main Eligibility Criteria/Study Population	Study Design	Intervention	Control
**Decrescendo,** **NCT04675827,** **Jules Bordet Institute**	- T ≥ 15 mm and ≤50 mm; N0 - ER-negative, PR-negative - Neoadjuvant therapy with paclitaxel or docetaxel + FDC SC trastuzumab and pertuzumab for 4 cycles	Phase 2, open-label, multicenter, non-randomized, sequential assignment	- RCB = 0: adjuvant pertuzumab + trastuzumab FDC SC for 14 cycles. - RCB 1: T-DM1 for 14 cycles. - RCB ≥ 2: anthracycline-based chemotherapy for 3–4 cycles + T-DM1 for 14 cycles.	N/A
**CompassHER2-pCR, NCT04266249,** **ECOG-ACRIN Cancer Research Group**	- cN0 if T2-3 - cN1-2 and T1-3 - Neoadjuvant therapy with paclitaxel or docetaxel + IV trastuzumab and pertuzumab for 4 cycles	Phase 2, open-label, multicenter, non-randomized, parallel assignment	- pCR: adjuvant trastuzumab and pertuzumab for 13 cycles - RD: adjuvant T-DM1 for 14 cycles +/− chemotherapy	N/A
**PHERGAIN-2,** **NCT04733118** **MedSIR**	- >5 to 25 mm and N0 by breast MRI	Phase 2 open-label, single-group assignment	- Neoadjuvant trastuzumab + pertuzumab FDC SC for 8 cycles - Based on pCR, 10 cycles of adjuvant therapy with trastuzumab + pertuzumab FDC SC or T-DM1. Chemo at the discretion of the physician	N/A
**TRAIN-3 study,** **NCT03820063,** **Borstkanker Onderzoek Groep**	- Stage II and III	Phase 2, multicenter, single-arm	Image-guided de-escalated neoadjuvant treatment	N/A
**NCT04419181,** **University of Rochester**	- Received 4 cycles of neoadjuvant docetaxel, carboplatin, trastuzumab and pertuzumab	Phase 2, open-label, non-randomized, parallel assignment	- pCR: adjuvant trastuzumab × 12 cycles - RD: adjuvant T-DM1 × 12 cycles +/− prior 2 more cycles of TCHP	
**DAPHNe,** **NCT03716180,** **Dana-Farber Cancer Institute**	- Stage 2 and 3 (T4d excluded) - Received neoadjuvant trastuzumab, pertuzumab and paclitaxel for 4 cycles	Phase 1, Open-label, single-group assignment	Adjuvant trastuzumab and pertuzumab w/o further chemotherapy if pCR achieved	
**IRIS-C/D,** **NCT04383275,** **Fudan University**	- ER and PR < 10%, T ≤ 2 cm - ER and/or PR ≥10% 1 cm < T ≤ 2 cm	Phase 2, open-label, single-group assignment	IRIS-C: Oral capecitabine for 4 cycles with standard trastuzumab for 1 yr IRIS-D: Oral vinorelbine for 4 cycles with standard trastuzumab for 1 yr	N/A
**IRIS,** **NCT04383275,** **Fudan University**	- For IRIS-A: ER and PR < 10%, T ≤ 2 cm OR ER and/or PR ≥ 10% 1 cm < T ≤ 2 cm - For IRIS-B: ER, PR ≥ 10% T ≤ 1 cm	Phase 2, open-label, single-group assignment	IRIS-A: capecitabine for 6 cycles with standard trastuzumab for 1 yr IRIS-B: endocrine therapy combined with standard trastuzumab for 1 yr	
**REaCT-HER-TIME,** **NCT04928261,** **Ottawa Hospital Research Institute**	- Stage I–III - pCR post neoadjuvant therapy	Phase 4, open-label, single-group assignment	Adjuvant trastuzumab for a total of 9 cycles every 3 weeks (or its equivalent if administered weekly), including the treatment received preoperatively	
**REaCT-LOW RISK HER2,** **NCT03705429,** **Lawson Health Research Institute**	Early-stage breast cancer for whom study regimens are being considered	Phase 3, open-label, multicenter, randomized	Docetaxel plus cyclophosphamide for 4 cycles and trastuzumab for 1 year	Weekly paclitaxel (× 12 weeks) and trastuzumab for 1 year

ClinicalTrials.gov searched on 11 March 2022, using the terms: “HER2-positive breast cancer” and “de-escalation”. CTC: circulating tumor cell; ER: estrogen receptor; FDC: fixed-dose combination; IV: intravenous; NCT: National Clinical Trial number (identification that ClinicalTrials.gov assigns a study when it is registered); pCR: pathologic complete response; PR: progesterone receptor; RCB: residual cancer burden; RD: residual disease; SC: subcutaneous; TCHP: docetaxel, carboplatin, trastuzumab and pertuzumab; Year: yr.
